# NanoLuc Luciferase – A Multifunctional Tool for High Throughput Antibody Screening

**DOI:** 10.3389/fphar.2016.00027

**Published:** 2016-02-18

**Authors:** Nicolas Boute, Peter Lowe, Sven Berger, Martine Malissard, Alain Robert, Michael Tesar

**Affiliations:** ^1^Molecular and Cellular Biology Unit, Institut de Recherche Pierre Fabre, Centre d’Immunologie Pierre FabreSaint-Julien-en-Genevois, France; ^2^Biochemistry Department, Institut de Recherche Pierre Fabre, Centre d’Immunologie Pierre FabreSaint-Julien-en Genevois, France

**Keywords:** NanoLuc^®^, luciferase, bioluminescence resonance energy transfer (BRET), homogeneous assay, affinity determination, expression analysis, Western blot, library screening, phage display

## Abstract

Based on the recent development of NanoLuc luciferase (Nluc), a small (19 kDa), highly stable, ATP independent, bioluminescent protein, an extremely robust and ultra high sensitivity screening system has been developed whereby primary hits of therapeutic antibodies and antibody fragments could be characterized and quantified without purification. This system is very versatile allowing cellular and solid phase ELISA but also homogeneous BRET based screening assays, relative affinity determinations with competition ELISA and direct Western blotting. The new Nluc protein fusion represents a “swiss army knife solution” for today and future high throughput antibody drug screenings.

## Introduction

Since the birth of recombinant protein technology a vast range of different protein fusion and protein tag systems (Terpe, 2003; Waugh, 2005) have been developed for screening and/or purification of biologics from various different libraries and expression hosts. These proved particularly useful for the detection of polypeptides that lack a constant region such as scFv (Bird et al., 1988). Concurrently automated high throughput screening platforms and miniaturization have constantly evolved (chip technology, fluidic systems) such that tag and reporter systems must cope with the required sensitivity, speed and economy on one side and promote early screening for favorable functionality and developability parameters (during early screening paths) on the other. To date, there is no tag or reporter protein that fulfills all parameters all at once. In most cases tags are combined in order to fulfill the basic requirements such as detection (Ullmann, 1984; Griep et al., 1999; Dammeyer et al., 2013) and purification (di Guan et al., 1988; Smith and Johnson, 1988; Smith et al., 1988; Cronan, 1990; Einhauer and Jungbauer, 2001; Schmidt and Skerra, 2007; Malpiedi et al., 2013). Therefore, the selection of such reporters and/or tags must be taken carefully as they are decisive for the selection and finally the quality of potential therapeutic antibody candidates.

For high throughput screening, homogenous immunoassays are ideal due to the absence of numerous incubation and washing steps. Due to signal-amplification the most sensitive reporter systems appear to be enzymes. Alkaline phosphatase (Kerschbaumer et al., 1997; Griep et al., 1999; Liu et al., 2013), beta-galactosidase (Ullmann, 1984) and *Gaussia* or Rluc (Venisnik et al., 2006; Han et al., 2013) have been successfully fused genetically and employed in screenings (Harper et al., 1997). However, those enzyme reporters increase substantially the size of the studied protein and the valency of the fusion construct. Alternatively, different fluorescent proteins such as GFP and YFP have been successfully applied as reporters (Cassimeris et al., 2013; Dammeyer et al., 2013) but still with a compromise of lower sensitivity, lower solubility and/or higher background of the fusion protein due to auto fluorescence.

Recently a novel luminescent protein, Nluc has been developed by Promega. Engineered by directed evolution from a deep-sea shrimp (*Oplophorus gracilirostris*) luciferase, the enzyme was concomitantly optimized with the identification of a novel substrate obtained by synthesis and screening of coelenterazine analogs (Hall et al., 2012). The resulting Nluc protein is a 19.1 kDa, monomeric, highly soluble and stable, ATP-independent enzyme. The novel substrate, furimazine, produces a glow type luminescence (half-life > 2 h) with a higher specific activity than Rluc or Fluc (Hall et al., 2012). The high luminescence intensity of Nluc, its high solubility and its small size (19.1 kDa) compared to Rluc (36 kDa) or to Fluc (61 kDa) indicate that Nluc could be a potent tool for *in vitro* protein–protein interaction assays.

Based on these new and favorable characteristics, we constructed several vectors that allow cloning of Nluc at the C-terminus of a scFv or the heavy chain of a complete IgG molecule. In parallel the assay conditions were optimized to further increase sensitivity. Finally this new reporter system was applied and tested by screening of scFv library pannings after subcloning of polyclonal outputs into the new Nluc fusion vector. The evaluation focused not only on its sensitivity and its adaptability to high throughput screenings but also on the following important first pass parameters that could be analyzed in parallel without purification step: (i) antigen and/or cell binding, (ii) productivity in procaryotic and eucaryotic expression systems and finally, (iii) affinity ranking. In addition the Nluc reporter showed its versatility in homogenous assay set-ups, competition ELISAs and Western blots.

## Materials and Methods

Protein targets used in this work are tyrosine kinase receptor family members (1–3) while target 4 is a nuclear protein.

### Generation of Nluc scFv and Antibody Fusions

The Nluc gene was amplified from vector pNL3.1 with primers: nLucMluS ATGGAAGCTCGACTTCCAGCTTG and nLucEcoAS CGCCAGAATGCGTTCGCACAGC using Phusion^®^ High-Fidelity DNA polymerase (NEB), and the PCR cycles, 98°C × 30 s × 1, followed by 30 cycles of 98°C × 5 s, 72°C × 20 s, followed by a final extension step of 72°C × 2 min. The resulting PCR product was subcloned into the phage display vector pPL101 replacing the gene III fusion protein. Nluc and vector DNA were digested with *Mlu* I and *Eco* RI purified and ligated with T4 DNA ligase. The ligation product was transformed in to chemically competent *Escherichia coli* Top10 cells (Invitrogen) according to the manufacturer instructions.

The Nluc gene was genetically fused to the C-terminal part of the constant domains of a human IgG1 by directed ligation (Lebedenko et al., 1991). Constant domains were amplified using the primers FwCH1-FcIgG1forNLuc TTAGGTCTCGCTAGCCCCCAGCAGCAAGA and RevCH1-FcIgG1forNLuc ATCTAGTCTGGTCTCTCGCCCTTGCCTGGGGACAGGCTCAGGCTCTTCTGGGTGT and the Nluc gene with the primers FwNLucFcIgG1fusion TTAGGTCTCTGGCGGCGGCGGCTCCATGGTCTTCACACTCGAAGATTTCGTTGGG and RevNLucFcIgG1fusion ATCTAGTCTGGTCTCGGATCCTTACGCCAGAATGCGTTCGCACAGCC using Phusion^®^ High-Fidelity DNA polymerase (NEB) following the instructions of the manufacturer. Purified PCR product were digested with BsaI-HF (NEB), ligated and cloned in the mammalian expression vector pCEP.

### Establishing Bioluminescence Assay

Purified NanoLuc (Promega), was distributed in a 96-well half-area white microplate (Costar, CLS3693) to a final concentration of 10 pmol/L in PBS; PBS, 0.1% BSA; 50% Nano-Glo assay buffer (Promega, N112B) in PBS or 50% Nano-Glo assay buffer, 0.1% BSA with two different dilutions (1/100 and 1/400 final) of furimazine (Nano-Glo^TM^ assay substrate, Promega, N113B). After a short centrifugation (500 rpm, 1 min) to gather reagent in the bottom of wells, luminescence was read (0.1 s/well, without filter) on a microplate reader (Mithras LB940, Berthold Technologies, Germany) after different incubation times at room temperature.

### Nluc Luminescence Titration

Purified Nluc and Nluc-fused antibodies were sequentially diluted in PBS-BSA 0.1% solutions. Ten microliters of each dilution were distributed in triplicates in a 96-well half area white microplate. Ten μL of furimazine diluted 200 times in PBS, 0.1% BSA were then added in each well. After a short centrifugation (500 rpm, 1 min), luminescence was read (0.1 s/well with or without a 530/25 nm filter) on a Berthold Mithras LB940 microplate reader.

### ELISA on Recombinant Protein

Ninety six well Immulon 2HB ELISA plates were coated with predetermined saturating concentrations of recombinant target protein in PBS 100 μL/well overnight at 4°C. The following day plates were saturated with 2% milk powder in PBS.

Random clones from panning to each target antigen cloned into pPL302 were inoculated in 100 μL 2× YT/Amp/0.1% Gluc and incubated for at least 6 h at 30°C with shaking. The scFv expression was induced by adding IPTG to a final concentration of 0.1 mmol/L and incubated overnight at 30°C with shaking. *E. coli* cells were lysed by adding an equal volume of 2× BBS/lysozyme buffer (24.7 mg/mL boric acid, 18.7 mg/mL NaCl, 1.48 mg/mL EDTA, 2.5 mg/mL lysozyme, adjusted to pH 8.0 with 10 mol/L NaOH) and following a 30 min incubation at room temperature were subsequently blocked with 1/5 culture volume of 12.5% milk powder, 0.05% Tween^®^ in PBS and were incubated for a further 30 min at room temperature. The cultures were then centrifuged for 5 min at 1800 *g* and 50 μL of supernatant added to precoated and saturated ELISA plates. Between each incubation step three washes with PBS, 0.05% Tween^®^ and three washes with PBS were performed. The scFv binding was detected by ELISA using the anti-pIII antibody pSKAN3 (Mobitech GmbH, Göttingen) and an HRP-labeled anti-mouse IgG A-4416 (Sigma-Aldrich, Saint-Quentin Fallavier) detection antibody with TMB ELISA peroxidase substrate (Interchim, Montlucon). Nluc labeled scFv were cultured extracted and saturated in the same manner, following a 1 h incubation step with target antigen, wells were washed as previously described and 50 μL of Nano-Glo^®^ substrate (Promega, N1120) diluted 1/400 in PBS was added. Luminescence was measured with a Mithras LB 940 (Berthold Technologies) with a read time of 0.1 s.

### Cell ELISA

Wild type and target-over-expressing Chinese Hamster Ovary (CHO) cells were seeded at 20000 cell/well in 96-well white microplate (CulturPlate, PerkinElmer) and cultured 2 days in Dulbecco’s Modified Eagle’s Medium (DMEM)-F12 supplemented with 5% FBS. After culture medium removal, cells were washed twice with phosphate-buffered saline (PBS). Wells were then saturated with a PBS-gelatine 0.5% solution 5 h at room temperature. After washing, cells were incubated with anti-target scFv-Nluc extracts or control 1 h at room temperature. After three washes with PBS, 50 μL of Nano-Glo^TM^ assay substrate (1/400 dilution in PBS, 0.1% BSA) was added and luminescence was read (0.1 s/well, without filter) on a Berthold Mithras LB940 microplate reader.

### Alexa488 Labeling of Recombinant Targets

Purified recombinant targets were labeled with Alexa Fluor 488 (Alex Fluor 488 carboxylic acid succinimidyl ester, Molecular Probes, A20000). Briefly, the recombinant target solution was first dialyzed overnight at 4°C against 0.1 M sodium bicarbonate pH 9.0 buffer. After dialysis, Alexa Fluor 488 (10 mg/ml in DMF) was added (dye-to-protein ratio of 20/1, mol/mol) and the mixture was incubated in the dark, 1 h at room temperature under continuous mixing. The reaction was stopped by dialysis overnight in the dark against phosphate buffer saline pH 7.4. The mixture was dialyzed twice. The protein concentration was determined by BCA assay. The labeled targets were further characterized by SDS-PAGE electrophoresis on 4–12% gradient gel under reducing and non-reducing conditions. The gel was stained with Coomassie Blue. The A488 Fluor labeling was assessed by reading the gel with Molecular Imager^®^ VersaDoc^TM^ MP Imaging System (Biorad). Alexa488-labeled recombinant targets were stored sterile at 4°C, protected from light.

### Homogenous BRET Assays

Two different configurations were used for homogenous BRET assays. In the first configuration we used target-Fc domain fusion proteins (1 μg/mL final) which were incubated with anti-target or control scFv-Nluc with or without a (Fab’)2 anti-hIgG-PE (phycoerythrin; Beckman Coulter, IM0550) diluted 1/1000 final in a white 96-well white microplate. After a 15 min incubation at room temperature, furimazine was added in all wells and BRET was immediately measured on a Berthold Mithras LB940 microplate reader by light-emission acquisition at 485 and 568 nm. In the second configuration, the BRET ratio was measured by comparison of light emitted by wells containing only anti-target or control scFv-Nluc versus wells containing anti-target or control scFv-Nluc and the target chemically labeled with Alexa488. In all configurations specific BRET signal (net BRET) expressed in milliBRET Unit (mBU) corresponds to the ratio of the light emitted by acceptors [Alexa488 (530 nm) or PE (568 nm) over donor Nluc (480 nm)] minus the same ratio measured on wells containing only the donor multiplied by 1000 (Angers et al., 2000; Boute et al., 2001).

### Binding Competition ELISA

Wells of a 96 well white flat bottom polystyrene High Bind microplate (Costar) were coated overnight at 4°C with recombinant target protein (0.2 μg/mL in PBS) followed by a saturation step of at least 2 h at 37°C with gelatine 0.5% in PBS. NanoLuc fused antibody 1 nmol/L was then added in each well simultaneously with various amounts of competitors or controls. After 1 h incubation and three washing steps, 50 μL of Nano-Glo^TM^ assay substrate (1/400 dilution in PBS/BSA 0.1%) was added and luminescence read (0.1 s/well) on a Berthold Mithras LB940 microplate reader.

### Western Blot

Briefly, commercial recombinant His tagged target 2 and Fc tagged target 3 extracellular domains (0.1 μg per lane) were migrated heated, non-reduced, on 4–15% precast polyacrylamide gel (Bio-Rad, Criterion TGX 567-1084) according to manufacturer’s protocol. Proteins were then transferred on nitrocellulose membranes using Trans-Blot^®^ Turbo^TM^ Midi Nitrocellulose Transfer Packs (2.5 A, 25 V, 7 min). After a 1 h, room temperature saturation step in Tris buffered saline, 0.05% Tween 20, 1% milk (TBS-T 1% milk), membranes were incubated overnight at 4°C with either a naked or a Nluc-fused anti-target 2 antibody (1 μg/mL), a 1/50 diluted periplasmic extract of Nluc fused-anti-target 2 scFv or Nluc fused-anti-target 3 scFv. After three washes in TBS-T, the membrane labeled with naked anti-target 3 antibody was incubated (1 h) with an anti-human kappa light chain-HRP secondary antibody (1/10000 in TBS-T 1% milk; Sigma, A7164). After three additional washes, the labeling was revealed with ECL blotting substrate (Pierce, 32106) on ECL film. In parallel, membranes labeled with Nluc fused scFv or antibody were washed three times with TBS-T and then revealed with furimazine (1/1000 in PBS, 0.1% BSA; Nano-GloTM assay substrate, Promega, N113B) on ECL film.

### Software

Analyses were carried out with GraphPad Prism6.

## Results

### Strategy and Constructs

For the evaluation of the Nluc Luciferase (Nluc) as a versatile reporter system five different antibodies have been used as either scFv Nluc fusions or IgG Nluc fusions or both. All five antibodies were directed against different antigens. The antibodies were applied in different experimental settings for proof-of-concept and to demonstrate the versatility of the Nluc as a reporter system.

Initially experimental conditions were optimized in order to obtain the highest sensitivity from the system. In a second step the Nluc reporter system was challenged in standard antibody screening procedures including (a) high throughput primary screens that could be easily adapted to automation (e.g., ELISA and homogeneous assay), (b) antibody competition set-up for epitope grouping or for the selection of optimized antibody variants (e.g., humanizations or affinity maturations) and finally c) the selection of antibodies that could be used in specific applications (e.g., Western blot).

### Optimization of Nluc Luciferase Assay Conditions

**FIGURE 1:**
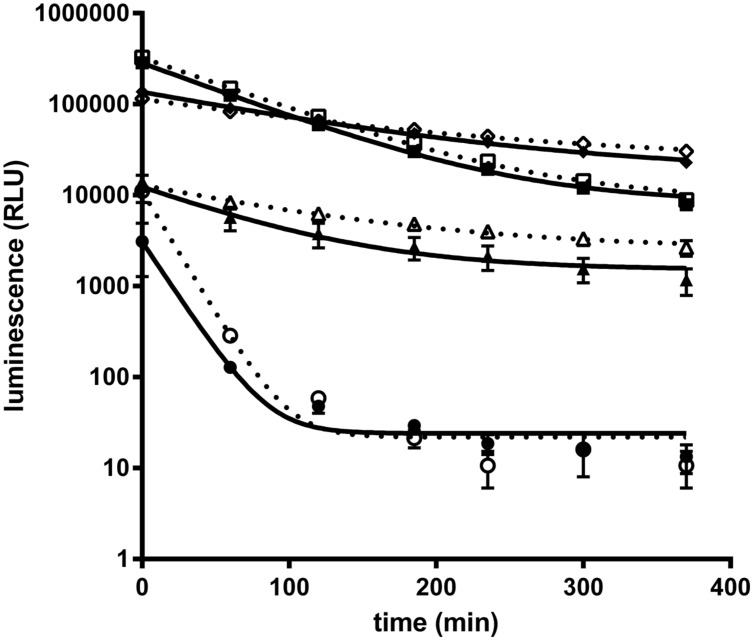
**Purified NanoLuc glow kinetic in different buffer conditions.** Luminescence signal of purified Nanoluc (10 pmol/L) was measured at several time points in a 20 μL final volume of several buffer conditions : PBS (circle), PBS, 0.1% BSA (square); 50% Nano-Glo assay buffer (Promega, N112B), 50% PBS (triangle) or 50% Nano-Glo assay buffer 50% PBS, 0.1% BSA (diamond) with 1/100 (white) or 1/400 (black) furimazine final dilution. Graphs of a representative experiment, each experimental point was performed in triplicate, and bars correspond to standard deviation (SD).

Recently a kit to detect Nluc activity comprising the Nano-Glo^®^ luciferase substrate (furimazine) and a lysis/reaction buffer has been commercialized (Promega). However, to adapt Nluc for antibody screening on protein antigens and whole cells the buffer conditions for the Nluc needed to be optimized. Especially, the presence of detergent in the reaction buffer as recommended by the supplier is not applicable for real time experiments on live cells. For the optimizations of assay conditions purified Nluc was used. As a starting point Nluc (10 pmol/L) performance in PBS showed a weak luminescence intensity and a fast decay of the signal compared to the signal obtained with the commercially supplied buffer (half-life of 17 min vs. 80 min; **Figure 1**). We found that through the addition of 0.1% BSA the Nluc luminescence signal strongly increased and even exceeded the signal of the commercial buffer by up to 25 fold. In addition the half-life increased from 17 to 60 min but still did not match that obtained with the commercially supplied buffer conditions (60 vs. 80 min). The difference in half-life is not the result of furimazine limitation since a similar half-life was obtained in PBS, 0.1% BSA when the Nluc substrate was diluted 400 times instead of 100 times as recommended by the supplier. Moreover, the addition of 0.1% BSA to Promega’s buffer conditions also greatly increased Nluc luminescence (up to 10-fold) reaching about half of the signal obtained for PBS supplemented with 0.1% BSA and an extended half-life of 100 min. Based on those results all further experiments were carried out in PBS supplemented with 0.1% BSA.

### Nluc scFv Fusions for High Throughput Primary Screening

**FIGURE 2:**
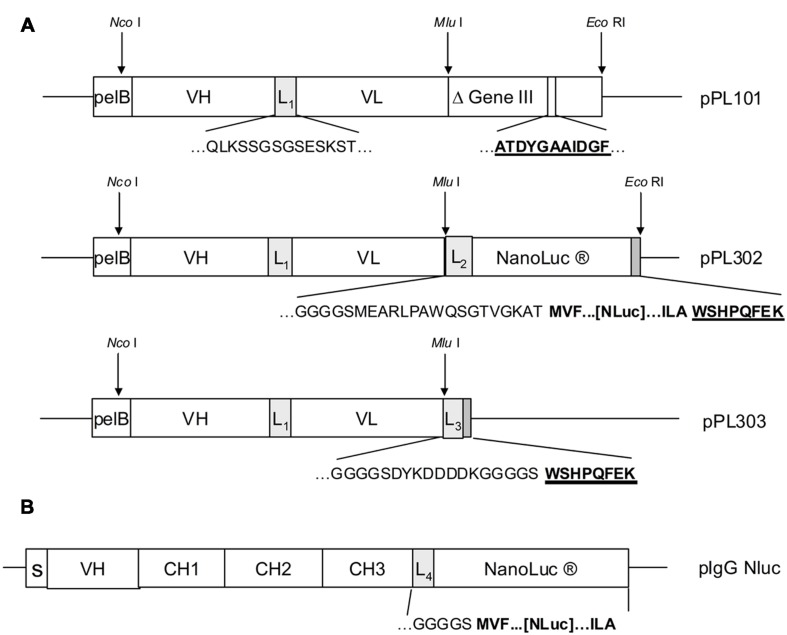
**(A)** Antibody libraries were created by cloning of assembled VH and VL genes after restriction digest with *Nco* I and *Mlu* I into phagemid vector pPL101. Cloned scFv were preceded by the pelB signal sequence and VH and VL genes were joined by a linker sequence (L1). The scFv was finally fused to the “short”(Δ) M13 gene III (corresponding to amino acids 251–406). After panning, the enriched phagemid population was digested by *Nco* I and *Mlu* I and subcloned into the expression vectors pPL302 and pPL303. Deduced amino acid sequences are shown for the most relevant fusion sites including linker sequences **L1**(QLKSSGSGSESKST), **L2**(GGGGSMEARLPAWQSGTVGKAT), **L3**(GGGSCYKDDDDKGGGGS), as well as for the anti-gIII (ATDYGAAIDGF) and the Strep-tag^TM^ epitope (WSHPQFEK). **(B)** For the pIgG Nluc vector the NanoLuc gene was fused downstream to the heavy chain IgG1 CH3 gene domain including the linker sequence L4. Deduced amino acid sequences at the fusion site are shown for the linker L4 (GKGGGGS) and three N- and C-terminal amino acids of NanoLuc (MVF…[Nluc]…ILA). Genes for the heavy chain variable region, the constant regions and the signal peptide are depicted as VH, CH1-3 and S.

In order to demonstrate that Nluc can be used as a reporter system and its small size (171 amino acids) neither compromises efficient bacterial production nor the function of a fusion partner, a scFv format has been chosen in a first application. For that purpose a Nluc expression vector was constructed which allowed the C-terminal fusion of single scFv genes or scFv pools to the N-terminus of the Nluc reporter gene *via* a 24 aa linker sequence derived from Promega vector pNL3.1 (**Figure 2A**) For direct comparison and calibration of hit rates the detection *via* anti-pIII (Tesar et al., 1995; Beckmann et al., 1998; Mersmann et al., 1998) and anti-Strep-tag^®^ II (Knappik and Brundiers, 2009) was used. Five distinct immune phage libraries generated in-house were subjected to three rounds of a standard phage panning on immobilized protein (Hoogenboom et al., 1998) and analyzed for specific scFvs directly expressed from the phagemid vector pPL101 *via* anti-pIII or, after subcloning into the two different expression vectors pPL302 via Nluc or pPL303 (**Figure 2A**), *via* an anti-Strep Tag II (Knappik and Brundiers, 2009). Primary screenings of the five different panning outputs were performed by ELISA with directly coated antigen and single chain-containing periplasmic *E. coli* extracts. Expression of scFv-Nluc was directly evaluated by measuring luminescence of culture supernatants or periplasmic extracts (**Table 1**). Binding ELISA was performed on purified antigen. An irrelevant antigen was coated at a saturating concentration to serve as a negative control for binding. The scFv Nluc fusion binding was directly measured after the scFv binding step followed by three washes, a secondary antibody was not required. As shown in **Table 1** comparable numbers of positive ELISA wells (hits) were obtained for all three detection systems and four different libraries except for target 3 library, L1, which resulted in approximately 40% lower hit-rate for the Nluc reporter system vector, possibly due to a bias in the sub cloning procedure. Hits for the Nluc reporter system were in most cases 100-fold above background and corresponding expression judged as positive for values above 10000 RLU. Despite the similarities of positive hit rate observed with the different detection strategies, sequence analysis was not performed at this stage and thus identified antibodies may not be the same between methods.

**Table 1:** Primary screening of panning outputs by ELISA.

	Hit Rate [%]^a^ and Expression Check [%]^b^ of Libraries (L1 – L5)				
Detection (vector): Antigen (libraries)	Target 3 (L1)	Target 1 (L2)	Target 3 (L3)	Target 4 (L4)	Target 4 (L5)
Anti-pIII^a^ (pPL101)	71	92	13	90	90
Anti-strep tag II^a^ (pPL303)	69	83	11	87	91
Nluc^a^ (pPL302)	32	80	10	87	98
Expression check^b^	93	100	98	nd	nd

*^a^Definition of “Hit”: signal > 5-fold over background.*

*^b^Definition of Expression: >1.0E+04 RLU; only measured for scFvs expressed from pPL302.*

*nd, not determined.*

### Luminescence Measurement for Estimation of Ab-Nluc or scFv-Nluc Fusion Concentration

**FIGURE 3:**
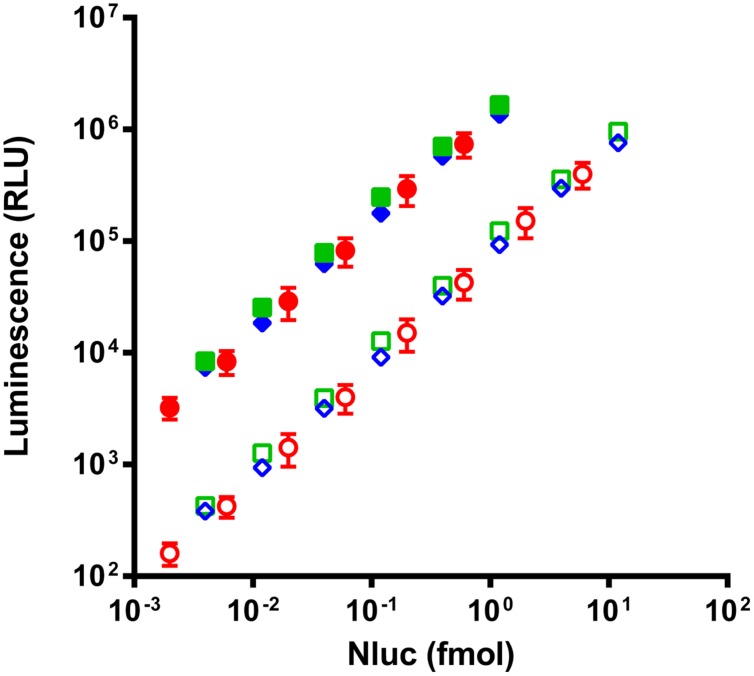
**Comparison of luminescence intensity of purified Nluc (circles) and two representatives of the four Nluc fused antibodies (squares and diamonds) read without filter (filled) or with a 530 nm filter (empty).** Antibody concentrations are corrected in equivalent Nluc as each antibody is linked to two Nluc. Each experimental point represents the means of three independent experiments made in triplicates, bars correspond to SD.

Due to the inherent difficulty to quantitate scFvs we chose to validate the quantification of fusion proteins via Nluc with complete antibodies for which alternative dosing strategies were readily available. Four different monoclonal antibodies in the IgG1 format were fused to Nluc (mAb-Nluc1 to 4) and compared to the luminescence signal of purified Nluc. To this end a new vector was designed and the Nluc gene fused to C-terminal end of the CH3 domain *via* a flexible linker (**Figure 2B**). IgGs were expressed in transiently transfected CHO EBNA cells, purified and further characterized by SDS-PAGE and size exclusion chromatography. All IgGs showed a monomeric fraction of >95% and no instability of the heavy chain-Nluc fusion was observed (data not shown). As expected (Hall et al., 2012; Zhang et al., 2013), a strong linearity was observed between the Nluc concentration and the luminescence signal (**Figure 3**). Non-linearity can occur with high enzyme level (>10 fmol/well) most likely due to lack of substrate or luminescence signal too close of reader saturation limit. After calculating equimolar Nluc concentrations for each of the IgG Nluc-fusion proteins results can be superimposed to the corresponding concentrations of purified Nluc. Moreover, the dynamic range can be adjusted by the use of a filter to avoid saturation of the photomultiplier for high Nluc concentrations even with a 0.1 s reading time. Without the use of a filter, saturation of our reader was observed when concentrations exceeded 4 fmol per well (i.e., 200 pmol/L in 20 μL), but with a 530 nm filter readings can be made in excess of 10 fmol per well. However, for higher Nanoluc concentrations, furimazine can become limiting and thus we would recommend sample dilution.

### NanoLuc Fusion Proteins in a Homogeneous Assay

For high throughput screening, homogenous assays are the best solution as they are faster, simpler, and well suited to automation. BRET (Couturier and Deprez, 2012) allows the study of protein–protein interactions by detection of the resonance energy transfer between a bioluminescent protein such as a luciferase and a compatible fluorescent acceptor coupled or fused to proteins of interest (Xu et al., 1999; Angers et al., 2000). Thus the binding of a scFv-Nluc to its target could be detected if the target is directly or indirectly labeled with a convenient fluorescent acceptor. The Nluc emission spectrum shows a maximum emission at 460 nm (Hall et al., 2012) which is compatible with the excitation profiles of various fluorophores such as Alexa-Fluor-488, phycoerythrin or YFP.

**FIGURE 4:**
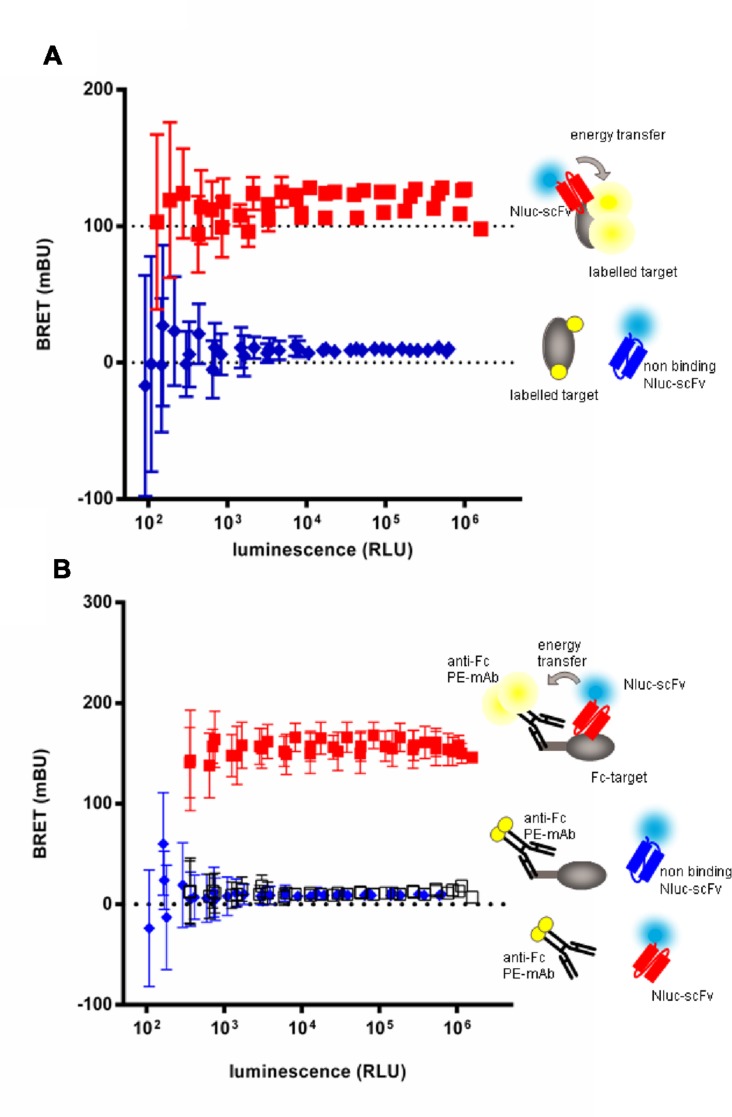
**Evaluation of homogenous BRET screening assay for detection of scFv-Nluc binders on a directly (A) or indirectly labeled target (B).** BRET signal measurement of increasing amounts of specific (squares) or aspecific (diamonds) Nluc fused scFv incubated either with a Alexa-fluor488 covalently labeled target **(A)** or an indirectly labeled target-Fc fusion with an (Fab’)2 anti-human Fc coupled to phycoerythrin **(B)**. BRET signal of specific scFv-Nluc incubated with an (Fab’)2 anti-human Fc coupled to phycoerythrin in absence of target is represented with empty square. Experimental points are means of triplicates of three independent experiments, bars are SD.

In order to set up the homogeneous assay, labeling conditions were tested with the extracellular domain of target 3 or its Fc-fusion derivative. The target 3 antigen was either directly coupled to Alexa-Fluor-488 (**Figure 4A**) or, in case of the Fc-fusion derivative, indirectly by an anti-human IgG(Fc) Fab’2 coupled to phycoerythrin (PE; **Figure 4B**). In a first step, the efficacy of BRET was tested with a reference anti-target 3 scFv-Nluc in dose response to determine the range of scFv-Nluc expression level allowing detection of positive hits. Anti-target 2 scFv-Nluc served as a negative control. **Figures 4A,B** show a robust BRET signal of 117.3 ± 10.6 and 156.3 ± 6.0 mBU, respectively, which was stable over a wide range of luminescence (from 10^3^ to 1.6 × 10^6^ RLU). This was expected as BRET signal is ratiometric, the same BRET signal will be obtained as long as the labeled antigen is in excess and the quantity of unbound scFv-Nluc is low. Both labeling procedures of the antigen were equally efficient with a low unspecific signal of 9.2 ± 2.4 and 9.5 ± 1.3 mBU, respectively. Moreover, a background BRET signal of 11.4 ± 2.5 mBU was obtained with the positive control scFv-Nluc and the anti-human IgG(Fc) Fab’2-PE in absence of target-Fc showing that no unspecific energy transfer occurred due to a direct binding of the labeled Fab’2 to scFv-Nluc. However, reading conditions under 1000 RLU resulted in significant increase of standard deviation and should not be taken into account.

**FIGURE 5:**
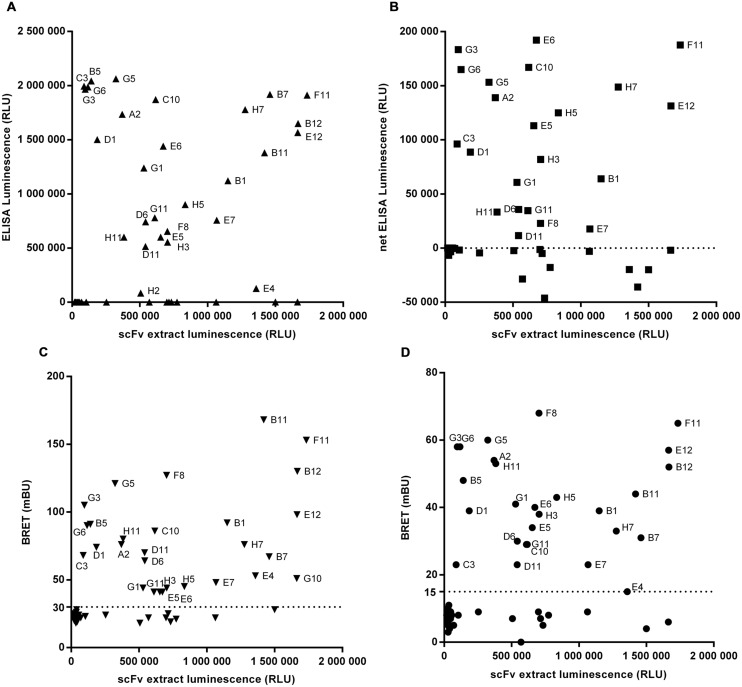
**Screening of 92 scFv-Nluc derived from library L3 panned against target 3 using either ELISA on recombinant protein (A), on cells (B) or the BRET assays with direct (C) or indirect (D) labeling of the target.** For all graphs, the x-axis corresponds to the luminescence of a sample of scFv extract before experiment indicating the relative quantity of each scFv. For cellular ELISA, results are the difference between the signal with CHO cells stably expressing target 3 and the signal of untransfected CHO. The dotted lines correspond to the selected threshold for positive clones.

For proof-of-concept, library output cloned into the Nluc vector pPL302 were produced and after expression level evaluation by luminescence reading, crude extracts were directly screened with BRET-based homogeneous assays using direct and indirectly labeled antigen. Results obtained were compared to those of recombinant protein or cellular ELISA. **Figure 5** represents the re-screening of 92 scFv-Nluc derived from library L3 panned against target 3 using either ELISA (**Figures 5A,B**) or the BRET assays (**Figures 5C,D**). For the ELISA assays, plates were coated with recombinant target 3-Fc-fusion protein or CHO cells stably over-expressing human target 3. Untransfected CHO served as a negative control. For the BRET assays, target 3 covalently labeled with Alexa-Fluor-488 or its Fc-fusion derivative labeled with Fab’2 anti-IgG-Phycoerythrin was used. Amongst these 92 scFv-Nluc products, 84 showed a sufficient expression level for accurate screening. ELISA on recombinant target 3 and BRET assays allowed the recovery of 27 positive clones while cellular ELISA confirmed only 22 of them (**Figures 5A,B**). Four of the five missing clones (B5, B7, B11, and B12) were not taken into consideration due to higher signal on the control CHO. The fifth clone (E5) already showed weak binding in protein ELISA. It is also worth mentioning that in BRET assay with Alexa-Fluor488 labeled target 3, an additional positive hit was detected (G10) whereas in ELISA on recombinant protein one additional weakly binding clone (H2) was included in the list of positives. However, the overall similarity of positive hits measured in ELISA and the homogeneous assays demonstrates the robustness and flexibility of Nluc-fusion protein screenings.

### ScFv and IgG Nluc Fusion in Competition ELISA

**FIGURE 6:**
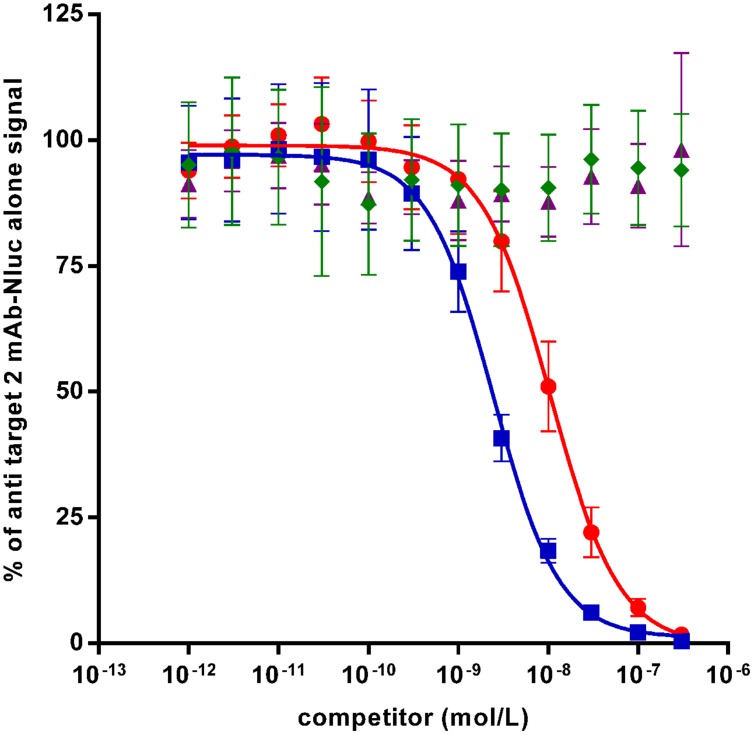
**Binding competition ELISA on recombinant target 2 between a Nluc-fused anti-target 2 mAb (1 nmol/L) and specific competitors [mAb (squares) and scFv (circles) or irrelevant antibodies mAb (diamonds) and scFv (triangles)].** Each experimental point is the mean of three independent experiments performed in triplicates, bars correspond to SEM.

In case of affinity maturations, humanizations and epitope mappings, competition assays are often used for screening of variants which have superior or different characteristics compared to the parental antibody. In order to test the Nluc reporter system for such applications, a competition assay has been set up with anti-target 2 mAb-Nluc fusion for proof-of-concept. As shown in **Figure 6** untagged anti target 2 mAb prevented the binding of 1 nmol/L of its Nluc-fusion in a dose-dependant fashion with an IC_50_ of 2.5 nmol/L while an irrelevant antibody showed no competition. The untagged scFv format of the anti-target 2 mAb was also able to compete with 1 nmol/L of the IgG Nluc-fusion but with an IC_50_ of 16.2 nmol/L. This higher IC_50_ most likely reflects the avidity of the IgG compared to the monovalent scFv.

### ScFv and IgG Nluc Fusion for Western Blotting

**FIGURE 7:**
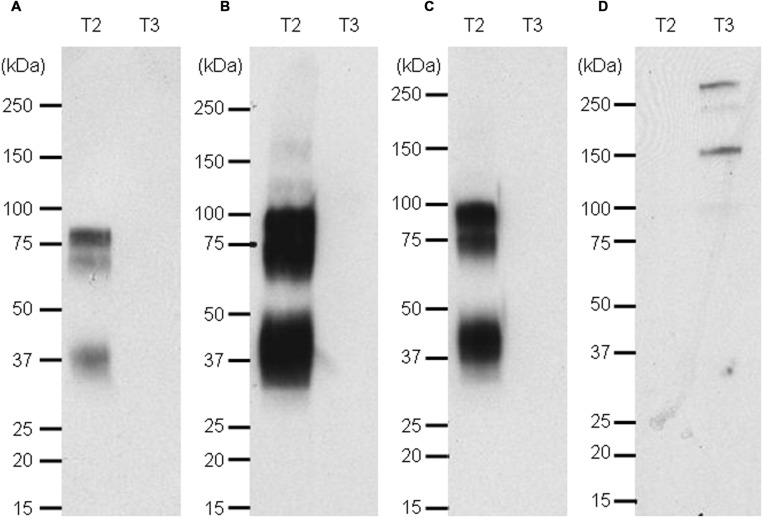
**Western blot of targets 2 and 3 revealed with naked anti target 2 mAb and an anti-human kappa light chain-HRP secondary antibody (A), the Nluc fused to anti-target 2 antibody (B), the scFv-Nluc version of the anti-target 2 antibody (C) and an anti-target 3 scFv-Nluc (D).** Membrane A labeling was revealed with ECL blotting substrate, while membranes **(B–D)** labeling was revealed with furimazine diluted 1000 fold in PBS, 0.1% BSA.

For a rapid screening of antibodies which can be used in Western blotting, the Nluc reporter system was applied for detection of recombinant targets 2 and 3 with specific antibodies. A band at the expected size of 80 kDa was detected for recombinant target 2 when the antibody was used as IgG (**Figure 7B**, lane T2) or scFv fused to Nluc (**Figure 7C**, lane T2) while no signal was detected on recombinant target 3 Fc fusion (**Figures 7B,C**, lanes T3). The same protein pattern was obtained with the untagged anti-target 2 antibody and standard peroxidase-staining although to a much weaker extent (**Figure 7A**, lane T2). One fainter additional band at 37 kDa could be observed most likely due to a degradation product of the recombinant target 2 protein. The target 3-Fc fusion protein could be detected at 140 kDa with the anti-target 3 Nluc-scFv. (**Figure 7D**, lane T3) An even higher migrating band at >250 kDa is most likely due to a portion of dimers.

## Discussion

The high number of binders to evaluate after a phage panning campaign requires efficient and fast methods of screening for the properties desired of hits. Nluc by its robustness, its small size and its bright luminescence activity showed that it could be a flexible and powerful tool for high throughput screening of panning outputs. We have shown that scFv fused Nluc allows the reduction of experimental steps, a low background, a high signal to noise and the possibility to establish homogenous assay all of which are key points for HTS. The strong intensity and the duration of the signal permit the detection of small quantities of products and thus the miniaturization of the assay volume, suitable for 384 and 1536 well plate format. We found that the high intensity of the signal permitted reduced reading times per well and that the long half-life of the signal opens the possibility of stacking plates before reading. In our assay, the scFv-Nluc were produced from only 120 μL of bacterial culture, although this volume was still in vast excess compared to the quantity actually needed to perform the individual analyses. Comparison of the luminescence from mAbs fused to Nluc and purified Nluc alone demonstrated that the fusion to a protein did not interfere with Nluc activity. This property allows the semi quantitative evaluation of scFv-Nluc expression levels an important and typically inaccessible value required to validate binding ELISA signals. It should be mentioned that while the intensity of ELISA signal is proportional to the quantity of bound Nluc fusion protein, this is not the case for BRET signals. Indeed, as we have shown, the BRET signal was the same for a wide range of quantities of a given scFv-Nluc as long as the target labeled with the acceptor is not limiting. Moreover two scFv-Nluc which bind with the same affinity to the same target could give different BRET signals if they bind to different epitopes due to spatial separation effects of donor to acceptor. The distance between the energy donor (Nluc) and the fluorescence acceptor should be less than 10 nm, for energy transfer to occur; a labeling strategy that exceeds this criterion will potentially create false negatives. The labeling strategies already employed within this work demonstrate that the margin for successful signal transfer is relatively large and should be readily adaptable to the majority of situations. Thus BRET technology allows the establishment of fast homogeneous assays allowing a quick identification of binders but not an affinity ranking, alternatively ELISA combined to the estimation of scFv-Nluc concentration gives information of the relative affinity between binders but requires additional experimental steps.

We have shown, that the fusion of Nluc to antibodies or scFv allows the establishment of various assays for screening and characterization (Western blots, ELISA, BRET, and competition assays). But the spectrum of assays that could be developed is potentially even greater. For example, functional competition assays to detect binders preventing ligand/receptor interaction can be developed. The sensitivity of BRET assays may also be improved through the use of novel acceptors such as NanoBRET acceptor (Promega) that has a broadly separated emission spectrum (Machleidt et al., 2015).

The characterization of hits identified during the screening process with Nluc offers several other opportunities. Several authors have shown that Nluc luminescence can be easily detected with a bioluminescence dedicated microscope (Hall et al., 2012; Stacer et al., 2013), thus we can speculate that Nluc fused antibodies could also find use as cell or tissue labeling agents for cell microscopy or immunohistochemistry. Furthermore, Stacer et al. (2013) described the use of Nluc as a reporter for small animal *in vivo* imaging, Tran et al. (2013) describing a similar approach with an Nluc expressing Influenza reporter virus. Circulating levels of Nluc fusions could easily be determined by regular sampling of Nluc signal in serum samples, biodistribution could in turn be followed via a small animal *in vivo* imaging device. The emission spectra peak of 460 nm, results in a strong attenuation of the signal in deep tissues (De et al., 2009; Stacer et al., 2013) a problem that may be surmountable by a shift of the light emitted toward the red wavelength by generating a kind of BRET3 using compatible mutant red fluorescent protein such as mOrange or the NanoBRET acceptor as proposed by De et al. (2009) with the RLuc8 mutant of the Rluc. The robust enzymatic activity of Nluc does decrease in acidic conditions (Hall et al., 2012). Based on this property, we envisage its use to follow Ab-Nluc or scFv-Nluc binding and internalization within cells through the resultant pH dependant signal decrease, which will permit the selection of candidates for antibody drug conjugate (ADC) therapies.

In summary, the small size, high stability and signal intensity of the luminescence, make Nluc a reporter of choice when fused to whole antibodies or fragments to generate multipotent tools essential for library screening, antibody characterization and *in vitro* and *in vivo* assays generation. The results demonstrate that Nluc is a versatile tool for screening of scFv libraries and other antibody formats. It does not interfere with solubility, binding or functionality and, showed an unsurpassed sensitivity in different ELISA set ups, including cell ELISA. The Nluc could be used for rapid quantification of the fusion partner and hence for rapid affinity rankings or affinity determinations by SET and finally could be used in homogenous immunological assays for cost-effective high throughput screens.

## Author Contributions

NB carried out establishment of bioluminescence assays, cell ELISA, competition ELISA, and BRET assays. PL carried out phage display libraries setting, scFv-Nluc production and ELISA screening. SB and AR carried out the generation of constructs and production of recombinant targets and Nluc-fused antibodies MM carried out purification and labeling of recombinant targets and Western blots. MT participated in the design of experiments and coordinated the study. All authors contributed to data analysis and interpretation as well as manuscript writing.

## Conflict of Interest Statement

The authors declare that the research was conducted in the absence of any commercial or financial relationships that could be construed as a potential conflict of interest.

## Abbreviations

BRET: bioluminescence resonance energy transfer
Fluc: Firefly luciferase
GFP: green fluorescent protein
Nluc: NanoLuc luciferase
Rluc: *Renilla* luciferase
scFv: single chain antibody fragment
SET: solution equilibrium titration
YFP: yellow fluorescent protein
